# 

**Published:** 1833-07-01

**Authors:** 


### CONTENTS

OF THE

### MEDICO-CHIRURGICAL REVIEW,

No. XXXVII. JULY 1, 1833,

### REVIEWS.

I.

New Views of the Process of Defecation, and their Application to Diseases of the Stomach, &c. By JAMES O'BEIRNE, M. D. .. .. .. .. .. .. .. .. .. .. 1

II.

Essay on the Natural History, Origin, Composition, &c. of Mineral Waters. By Dr. GAIRDNER .. .. .. .. .. .. .. .. .. .. .. .. .. .. .. 28

II. (bis.)

A Treatise on the Venereal Disease and its Varieties. By WILLIAM WALLACE. Esq. 38

III.

PERICARDITIS.

I. M. Louis' Memoirs and Researches, Anatomical and Pathological, &c.
II. Clinique Medicale. Par M. ANDRAL. Second Edition .. .. .. .. .. 49

IV.

Researches on the Pathology and Treatment of some of the most important Diseases of Women. By ROBERT LEE, M. D. .. .. .. .. .. .. .. .. .. .. 58

V.

M. DUPUYTREN'S Clinical Lectures at the Hôtel Dieu .. .. .. .. .. .. .. 68

VI.

Mr. MAYO'S Outlines of Human Physiology.. .. .. .. .. .. .. .. .. .. .. 91

VII.

A Treatise on Diseases of the Urethra. By Mr. PHILLIPS.. .. .. .. .. .. .. 107

VIII.

The Black Death in the Fourteenth Century. By Dr. BABINGTON.. .. .. .. .. 121

IX.

Graphic Illustrations of Abortion and the Diseases of Menstruation. By Dr. GRANVILLE.. .. .. .. .. .. .. .. .. .. .. .. .. .. .. .. .. 130

X.

MORBID ANATOMY.

I. Anatomie Pathologique du Corps Humain. Par J. CRUVEILHIER, M.D.. .. ..

II. Illustrations of the Elementary Forms of Disease. By ROBT. CARSWELL, M.D. 137

### PERISCOPE.

### PART I.

### Spirit of English Periodical Literature.

1. Dr. Graves on the Treatment of various Diseases .. .. .. .. .. .. .. 145
Head-ache in Young Women .. .. .. .. .. .. .. .. .. .. .. .. 145
Exhibition of Opium in Enema .. .. .. .. .. .. .. .. .. .. .. 148
Dysphagia .. .. .. .. .. .. .. .. .. .. .. .. .. .. .. 149
2. The Whites and the Blacks, phrenologically considered .. .. .. .. .. .. 150
3. Development of the Head of Dr. Spurzheim.. .. .. .. .. .. .. .. .. 152
4. On the Pathology of Purpura .. .. .. .. .. .. .. .. .. .. .. .. 153
5. Restoration of Vision in Cases of Staphyloma and incurable Opacity of the Cornea.. .. .. .. .. .. .. .. .. .. .. .. .. .. .. .. 156
6. Abscess of the Kidney and Cerebellum .. .. .. .. .. .. .. .. .. .. 159
7. Salutarium in the Western Highlands .. .. .. .. .. .. .. .. .. .. 160
8. Obituary of Dr. Babington, with some Remarks.. .. .. .. .. .. .. .. 161
9. (Review)—On Hysteralgia, or Irritable Uterus. By Dr. Davis .. .. .. 162
10. (Review)—Observations on the present State of Pharmacy in Ireland. By Mr. Phelan.. .. .. .. .. .. .. .. .. .. .. .. .. .. .. .. 166
11. Discharge of Worms from various Parts of the Body. By Mr. Neilson .. .. 168
12. (Review)—Observations on the Testicles. By Professor Russell .. .. .. 169
13. Dr. Stokes on Gastritis and Dyspepsia .. .. .. .. .. .. .. .. .. .. 175
14. Mr. Robertson on Dry Cupping .. .. .. .. .. .. .. .. .. .. .. .. 178
15. The Medical Boards—Vaccine Board .. .. .. .. .. .. .. .. .. .. 180
16. The Factories' Regulation Bill .. .. .. .. .. .. .. .. .. .. .. 181
17. The Heliotrope; or, Pilgrim in Pursuit of Health .. .. .. .. .. .. 183
18. March of Intellect. Magnetic Extraction of Tic Douloureux—Method of Dissolving Calculus in the Bladder—Surgical Operations during Somnambulism, 184
19. Labour or Ennui? Which is the greater Evil? Factory Labour .. .. .. .. 185
20. Dr. Carswell on Carcinoma .. .. .. .. .. .. .. .. .. .. .. .. .. 186
21. Retention of Urine, from Imperforate Hymen .. .. .. .. .. .. .. .. 190
22. Hysteria fatal .. .. .. .. .. .. .. .. .. .. .. .. .. .. .. .. 191
23. Memorial of the London Physicians .. .. .. .. .. .. .. .. .. .. 192

### PART II.

### Spirit of the Foreign Periodicals.

24. Report of the Medical School in Egypt .. .. .. .. .. .. .. .. .. .. .. 193
25. Professor Roux on the Cause of Death after severe Injuries .. .. .. .. .. 195
26. Boiling Liquor Potassæ swallowed .. .. .. .. .. .. .. .. .. .. .. .. 195
27. Amputation at the Hip-joint .. .. .. .. .. .. .. .. .. .. .. .. .. 195
28. Chlorosis mistaken for Disease of the Heart.. .. .. .. .. .. .. .. .. 196
29. Variola after Vaccination.. .. .. .. .. .. .. .. .. .. .. .. .. .. 196
30. Turpentine in Sciatic Neuralgia .. .. .. .. .. .. .. .. .. .. .. .. 197
31. Reproduction of the Chrystalline Lens .. .. .. .. .. .. .. .. .. .. 197
32. Lisfranc's Treatment of Amaurosis.. .. .. .. .. .. .. .. .. .. .. 199
33. Obliteration of the Uterine Veins after Puerperal Fever .. .. .. .. .. 199
34. Observations on the Morbid Anatomy of the Viscera in Puerperal Fever.. .. 200
35. On Hydatids, and their Conversion into Tubercles .. .. .. .. .. .. .. 201
36. New Speculum Uteri—Cure of Sterility.. .. .. .. .. .. .. .. .. .. 202
37. Induction of Premature Labour by means of Sponge and by Puncture .. .. 202
38. Malignant Carbuncle in Italy.. .. .. .. .. .. .. .. .. .. .. .. .. 203
39. Extemporaneous Vesication .. .. .. .. .. .. .. .. .. .. .. .. .. 203
40. Remedies against Scrofula .. .. .. .. .. .. .. .. .. .. .. .. .. 203
41. Insanity in Italy.. .. .. .. .. .. .. .. .. .. .. .. .. .. .. .. 203
42. Two extraordinary Cases of Fasting .. .. .. .. .. .. .. .. .. .. .. 204
43. Extraordinary Case of Congenital Bulimia .. .. .. .. .. .. .. .. .. 206
44. Arteritis and Spontaneous Gangrene .. .. .. .. .. .. .. .. .. .. .. 207
45. Quinine successfully used in Tooth-ache .. .. .. .. .. .. .. .. .. .. 209
46. Preservation of Leeches by feeding them with Sugar .. .. .. .. .. .. 209

47. Gangrene of the Lungs .. 209

48. Sudden Death from Paralysis of the Lungs .. 210

49. Fatal Purpura Hæmorrhagica .. 210

50. Double Vision in one Eye .. 211

51. Medical Monomania .. 211

52. Fissures and Depressions in the Sculls of New-born Infants .. 211

53. Total Mortality from Cholera in France, 95,000 ! .. 212

54. On Pneumonia Hypostatica .. 212

55. Purulent Diathesis—Pericarditis .. 213

56. Anatomical Anomalies .. 213

57. M. Sedilot on Plica Polonica .. 214

58. Intestinal Entozoa .. 214

59. Amaurosis from Onanism in a Female .. 214

60. Ossification of the Retina .. 215

61. Aromatic Odour exhaled from the Arm .. 215

62. Chloride of Lime in Psora .. 215

63. Fatal Effects of a Tartar-Emetic Plaster .. 215

64. Leech-bite Hæmorrhage fatal .. 216

65. Kermes Mineral in Pneumonia .. 216

66. M. Pigeaux on the Sounds of the Heart .. 216

67. Neuralgia—Intermittent Cough .. 217

68. M. Dupuytren on Prolapsus of the Rectum .. 217

69. Researches on the Conium Maculatum. By Foderé .. 218

70. Dr. Cerioli on Hydro-Ferro-Cyanate of Quinine in Intermittent Diseases .. 219

71. Iron in Chlorotic Gastralgia .. 220

72. Singular Convulsive Disease .. 220

73. Indian Ophthalmia treated by Alum .. 221

74. Clot Bey on Cholera in Egypt .. 222

75. On Cyanosis .. 223

76. Cases of Pleuro-pneumonia treated by large Bleedings .. 225

77. On the Qualifications of a Physician. By M. Tiedeman .. 227

78. Remarkable Cures. By the late M. Dance—at the Hôtel Dieu.

1. Hypertrophy of the Heart .. 228

2. Complete Paraplegia .. 228

3. Chronic Inflammation of the Cervix Uteri .. 229

Institute of France—Academy of Sciences.

79. M. Dutrochet on the Mechanism of Respiration in Aquatic Insects .. 230
80. M. G. St. Hilaire on Hermaphrodism .. 231
81. On the Adulteration of Salt in France .. 231
82. Luminous Urine .. 232
83. Anatomy of the Medusa Marsupialis. By Dr. Edwards .. 232
84. On the Ascension of the Roots of some Plants .. 232
85. Report of the Academy on St. Hilaire's Work on Hermaphrodism .. 232
86. Effects of large Quantities of Quicksilver swallowed .. 233
87. M. Boussingault on the Thermal Waters of the Andes .. 234
88. M. Maingault on Tracheotomy .. 234
89. Double, or Bilobed Uterus—Disease of the Kidneys—Bilobed Bladder .. 234
90. M. Dubois on the Presentation of the Head in Labour .. 235
91. Epidemic Fever at Besancon .. 235
92. Singular Case of Paraplegia .. 236
93. Remarkable Variety of Hermaphrodism .. 237
94. Satyriasis following a Blow on the Head .. 238
95. Anomaly in the Arch of the Aorta .. 239
96. Two Cases of Œsophagotomy. By M. Begin .. 239
97. Death from Admission of Air into the Jugular Vein .. 241
98. Insalubrity of Paris .. 241
99. Imperfection in the Heart, without Cyanosis .. 242
100. Extirpation of a Diseased Ovary .. 242
101. Purulent Diathesis .. 242
102. External Discharge of Biliary Calculi .. 243
103. Treatment of Hooping-Cough and Measles by Antimonial Frictions .. 244

104. Majendie's Lectures on Cholera .. .. .. .. .. .. .. .. .. .. .. .. .. 244

105. Diptherite—tracheotomy .. .. .. .. .. .. .. .. .. .. .. .. .. 245

106. New Operation for Laryngo-pharyngeal Fistulæ .. .. .. .. .. .. .. 246

107. Curious Monstrosity—Germination .. .. .. .. .. .. .. .. .. .. 246

108. New Conjecture on the Cause of the Circulation .. .. .. .. .. .. .. 247

109. Organs of Circulation in the Crocodile.. .. .. .. .. .. .. .. .. .. 247

PART III.

### Clinical Review and Hospital Reports.

110. Surgical Report from the Glasgow Royal Infirmary. By Mr. Stirling .. .. 249
Foreign Body in the Trachea—Operation .. .. .. .. .. .. .. .. .. 249
Tumour in the Neck—Laryngotomy .. .. .. .. .. .. .. .. .. .. 250
Wound of Throat—Pharynx opened .. .. .. .. .. .. .. .. .. .. 251
Carcinoma Recti .. .. .. .. .. .. .. .. .. .. .. .. .. .. 252
Injuries of the Head.. .. .. .. .. .. .. .. .. .. .. .. .. .. 253
Excision of the Elbow-joint .. .. .. .. .. .. .. .. .. .. .. .. 255

111. Birmingham Infirmary.
Mr. Middlemore's Ophthalmic Report.. .. .. .. .. .. .. .. .. .. 257
Small-pox Pustule of the Cornea .. .. .. .. .. .. .. .. .. .. .. 257
Conical Cornea.. .. .. .. .. .. .. .. .. .. .. .. .. .. .. 258
Quina in Strumous Affections .. .. .. .. .. .. .. .. .. .. .. 258
Hydrophthalmia .. .. .. .. .. .. .. .. .. .. .. .. .. .. 259
Strychnia in Amaurosis .. .. .. .. .. .. .. .. .. .. .. .. .. 260

112. Bristol Infirmary.
Case of Osteo-Sarcoma, with Operation .. .. .. .. .. .. .. .. .. 261

113. Liverpool Northern Dispensary.
Case of Melanosis .. .. .. .. .. .. .. .. .. .. .. .. .. .. 263

114. Bridgewater Infirmary.
Fixing the Scapula in Cases of Dislocation .. .. .. .. .. .. .. .. 265

115. Rathkeale Dispensary.
Asphyxia of New-born Children .. .. .. .. .. .. .. .. .. .. .. 265
Hemeralopia .. .. .. .. .. .. .. .. .. .. .. .. .. .. .. 266
Iodine in Caries of the Vertebræ .. .. .. .. .. .. .. .. .. .. 266

116. Bristol Infirmary.
Inguinal Aneurism .. .. .. .. .. .. .. .. .. .. .. .. .. .. 267

117. Dublin-County Infirmary.
Mr. Crampton on Dislocation of the Shoulder .. .. .. .. .. .. .. 270

118. St. George's Hospital.

I. Two Cases of Morbid Growth from the Lower Jaw .. .. .. .. .. .. 274

II. Case of Morbid Growth, apparently fungoid, of the Antrum .. .. .. 277

III. Polypoid Growth from Æthmoid Cells.. .. .. .. .. .. .. .. .. 278

IV. Scrofulous Disease of Lower Jaw.. .. .. .. .. .. .. .. .. .. 279

119. Guy's Hospital.
Ulcers of the Face treated by Acids .. .. .. .. .. .. .. .. .. .. 279
Strumous Ulcers commencing in Ecthyma.. .. .. .. .. .. .. .. .. 280

120. London Hospital.
Osteo-Sarcoma—Removal of the Superior Maxillary and Malar Bones .. .. 281

121. Middlesex Hospital.

I. Sir C. Bell on Contraction of the Prepuce .. .. .. .. .. .. .. .. 282

II. Femoral Hernia, with double Hernial Sac .. .. .. .. .. .. .. .. 283

PART IV.

### Miscellanies.

1. Periodical Press .. .. .. .. .. .. .. .. .. .. .. .. .. .. .. 284

2. Remarkable Cessation of the Pulse in both Arms. By Dr. Fergusson .. .. 285

3. Cholera .. .. .. .. .. .. .. .. .. .. .. .. .. .. .. .. 285

4. Medical Politics—Apothecaries.. .. .. .. .. .. .. .. .. .. .. .. 286

Bibliographical Record.. .. .. .. .. .. .. .. .. .. .. .. .. 286

